# Lifestyle and Chemicals: Exploring Behavioral Habits Related to Endocrine Disruptor Exposure Among the General Population of Saudi Arabia

**DOI:** 10.7759/cureus.64392

**Published:** 2024-07-12

**Authors:** Dalal M Alabdulmohsen, Layan A AlDeaiji, Umar A Abdul Hai, Mohammed Y Ghazwani, Khalid M Alsulaim, Ryanh H Alanazi, Sarah S Alahmari, Njood O Omar, Ameera A Elfeky, Adnan M Almarzouq

**Affiliations:** 1 Internal Medicine Department, College of Medicine, King Faisal University, Hofuf, SAU; 2 Internal Medicine Department, College of Medicine, Qassim University, Unaizah, SAU; 3 Internal Medicine Department, College of Medicine, King Abdulaziz University, Jeddah, SAU; 4 Internal Medicine Department, College of Medicine, Jazan University, Jazan, SAU; 5 Internal Medicine Department, College of Medicine, Northern Border University, Arar, SAU; 6 Internal Medicine Department, College of Medicine, King Khalid University, Abha, SAU; 7 College of Clinical Pharmacy, Imam Abdulrahman Bin Faisal University, Dammam, SAU; 8 Internal Medicine Department, College of Medicine, Newgiza University, 6th of October City, EGY; 9 Endocrinology Department, Dr. Sulaiman Al-Habib Hospital, Al-Khobar, SAU

**Keywords:** environmental health, cross-sectional studies, awareness, saudi arabia, chemical exposure, preventive health services, endocrine disruptors

## Abstract

Background: Endocrine-disrupting chemicals (EDCs) interfere with hormonal systems, potentially causing metabolic, reproductive, and neurological issues, as well as hormone-related cancers. Found in everyday products, EDCs accumulate in body tissues over time, with adverse effects depending on the dose and duration of exposure. This study aims to explore behaviors related to EDC exposure among Saudi citizens to assess the need for further risk reduction interventions.

Methodology: This cross-sectional study employed a validated, self-administered online questionnaire to assess daily life behaviors associated with EDC exposure. A total of 563 participants were recruited using convenient sampling through online platforms.

Results: The study revealed that a significant majority of participants were aged 18-25 years (48.67%, n=274). On average, participants scored 32.78 out of a maximum of 60 for potential EDC exposure, with scores ranging from 13 to 54 points. The majority (85.26%, n=480) fell into the moderate potential exposure category, while a small minority (4.26%, n=24) exhibited high potential risk based on their reported daily habits, predominantly among male participants (95.83%, n=23). A significant majority (72.65%, n=409) indicated a likelihood of adopting lifestyle changes to reduce exposure to harmful substances.

Conclusion: This study reveals diverse behavioral patterns linked to endocrine disruptor exposure among the general population in Saudi Arabia. Interestingly, the participants showed a positive attitude and willingness to change their risky behaviors. These findings underscore the necessity for educational programs and public health campaigns aimed at addressing gaps in knowledge. Encouraging the public to adopt behaviors that reduce exposure is essential to minimizing the potential long-term effects of EDCs.

## Introduction

Endocrine-disrupting chemicals (EDCs) are natural or man-made chemicals that can interfere with the body's hormone system. These chemicals can mimic, block, or otherwise alter the way hormones work. EDCs are found in many everyday products, entering the human body through ingestion, inhalation, and skin absorption [1]. Research suggests a potential link between EDC exposure and various health problems, including potential disruptions to metabolic processes, reproductive function, and neurological development. Moreover, there are particular concerns about the association of EDCs with certain cancers that are hormone-related, such as breast, endometrium, and prostate cancers [2]. Common examples of these substances include bisphenol A (found in plastic food wrappers), phthalates (found in cosmetics), parabens (found in personal care products), and pesticides. Because EDCs can accumulate in body tissues over time, sometimes for years, it is possible that significant adverse effects might only occur after longer exposure or higher doses. This emphasizes the importance of dose and duration when considering how EDCs may affect human health [3].

Exposure to EDCs is unavoidable because of the widespread presence of these chemicals in our everyday environments. However, as it is dose-dependent, interventions to reduce exposure are worth trying. These interventions can be national (by governments and foundations) or personal (by lifestyle and behavior changes). The latter is particularly significant for the effective minimization of exposure [4]. A study by Kim et al. summarized a number of interventional studies on endocrine disruptors. These studies focused on a number of common endocrine-disrupting substances, including bisphenol A, phthalates, and parabens, which are extensively prevalent in the daily environment. Interventions covered in the study were behavior-based interventions. They included dietary changes, personal care product changes, and other in-home interventions that all aimed to reduce the amount of chemicals that the participants were exposed to. After only one month of these behavioral changes, concentrations of the tested EDCs in participants' urine significantly decreased [5].

In Saudi Arabia, several recent studies were conducted to assess EDC levels in food [6,7], water [8,9], and house dust [10]. These studies highlight the risk of EDCs, bring our attention to the issue, and encourage us to take further action in this regard. A study performed by Alkhalidi et al. measured Saudis' awareness of certain EDCs that can interfere with sex hormones. Their study covered females of reproductive age from Al-Jouf city and showed poor knowledge regarding the impact of these chemicals [11]. In this study, we aim to explore the behaviors related to increased EDC exposure in Saudi Arabia. The goal is to explore the current lifestyle choices of Saudi citizens and assess the need for further risk reduction interventions.

## Materials and methods

Research design and participants

This cross-sectional study, conducted from May to June of 2024 in Saudi Arabia, aimed to assess the lifestyles of the Saudi population regarding exposure to EDCs in the home environment. A convenient sampling technique was employed by recruiting participants through online platforms (WhatsApp and Telegram).

Sample size and inclusion/exclusion criteria

Based on the latest Saudi census data (2022, overall population: 32,175,224; Saudi citizens: 58.4%), an online sample size calculator determined a minimum of 385 participants for a 95% confidence level and a 5% margin of error and assuming a 50% prevalence rate [12]. To account for potential missing data, we sent the questionnaire to 600 potential participants. The study included Saudi residents of all ages. Non-Saudi residents, those who did not provide consent, or those who did not complete the questionnaire were excluded.

Questionnaire development and validation

A self-administered online questionnaire was developed to assess daily life behaviors related to EDC exposure. The questionnaire was informed by resources from the Health and Environment Alliance (HEAL)'s "Tips to Avoid Endocrine Disrupting Chemicals In and Around The Home" [13] and the Environmental Working Group (EWG)'s "Guide To Endocrine Disruptors" [14]. Each question began with "How often do you...?" and employed a five-point Likert scale to assess frequency (0-4 points). The questionnaire was translated into the Arabic language, reviewed by three experts, and validated for internal consistency using Cronbach's alpha (reliability coefficient=0.76).

Scoring system

Scoring reflected the potential impact of behaviors on EDC exposure. Behaviors associated with increased exposure received higher points for "always" (4 points) and lower points for "never" (0 points). Conversely, behaviors promoting lower exposure received higher points for "never" (4 points) and lower points for "always" (0 points).

With 15 questions, the total score ranged from 0 to 60. A higher score indicated a need for lifestyle modifications to reduce EDC exposure, while a lower score suggested adherence to recommended practices. For further analysis, participants were categorized based on their scores: 0-20 (potential low exposure), 21-40 (potential moderate exposure), and 41-60 (potential high exposure).

Data management and analysis

All data was organized in an Excel spreadsheet and analyzed using IBM SPSS Statistics for Windows, Version 27.0 (Released 2020; IBM Corp., Armonk, New York, United States). Descriptive statistics (frequencies and percentages) were used to characterize participant demographics and lifestyles. Chi-squared tests investigated potential associations between demographic characteristics and potential EDC exposure levels. A significance level of p<0.05 was adopted.

## Results

Demographics

A total of 563 participants from Saudi Arabia participated in the study, showing a response rate of 93.83%. The majority (48.67%, n=274) were aged 18-25 years old. Regarding education, most participants (43.87%, n=247) held a bachelor's degree. The central region had the highest response rate (37.48%, n=211). Gender distribution was nearly equal, with 52.40% (n=295) female participants. Table 1 shows a summary of participants' characteristics.

**Table 1:** Socio-demographic information of the participants (n=563). The data has been presented as frequencies (n) and percentages (%).

Socio-demographic information	Category	Frequency and proportion n (%)
Age in years	Less than 18	37 (6.57%)
	18-25	274 (48.67%)
	26-35	132 (23.45%)
	36-45	52 (9.24%)
	46-55	45 (7.99%)
	56-65	22 (3.91%)
	More than 65	1 (0.18%)
Gender	Female	295 (52.40%)
	Male	268 (47.60%)
Nationality	Saudi	563 (100.0%)
Area of residence	Central region	211 (37.48%)
	Southern region	121 (21.49%)
	Western region	91 (16.16%)
	Northern region	75 (13.32%)
	Eastern region	65 (11.55%)
Education level	Doctorate	2 (0.36%)
	Masters	22 (3.91%)
	Bachelor's	247 (43.87%)
	Diploma	62 (11.01%)
	High school	201 (35.70%)
	Middle school	28 (4.97%)
	Elementary school	1 (0.18%)

Practices related to EDCs

Around 65% (n=366) of participants reported always washing and peeling fruits and vegetables before eating. However, only 14% (n=79) reported consuming organic food regularly. Interestingly, while 41% (n=231) never heated food in plastic containers using a microwave, 36% (n=203) used non-stick cookware consistently. Additionally, take-out food consumption from plastic or cardboard containers was frequent among 53% (n=298) of participants. A considerable proportion of participants (50%, n=282, and 45%, n=254, respectively) always used plastic water bottles and personal care products without checking labels for potential EDCs. On a positive note, 47% (n=265) reported daily home ventilation, and nearly half (49%, n=276) cleaned dust with wet cloths. Additionally, a third (34%, n=191) rarely used pesticides at home or in their gardens. Only 23% (n=130) used air fresheners and fragrances daily, and 46.5% (n=262) confirmed they always take the thermal receipt with them after purchases. Table 2 and Table 3 show the frequencies and proportions of the participants' responses.

**Table 2:** Behaviors that reduce exposure to harmful chemicals. The data has been presented as frequencies (n) and percentages (%).

Question	Frequency n(%)				
How often do you…	Always	Often	Sometimes	Rarely	Never
wash new clothes, kitchen equipment, and other products before use?	280 (49.73%)	127 (22.56%)	104 (18.47%)	39 (6.93%)	13 (2.31%)
ventilate the house for 10 minutes 1-2 times a day?	265 (47.07%)	118 (20.96%)	106 (18.83%)	51 (9.06%)	23 (4.09%)
wash and peel fruit and vegetables before eating?	366 (65.01%)	104 (18.47%)	67 (11.90%)	23 (4.09%)	3 (0.53%)
use damp cloths while cleaning dust regularly?	276 (49.02%)	157 (27.89%)	99 (17.58%)	19 (3.37%)	12 (2.13%)
consume organic food?	79 (14.03%)	107 (19.01%)	236 (41.92%)	108 (19.18%)	33 (5.86%)
wash hands with soap or water before eating?	259 (46.00%)	153 (27.18%)	124 (22.02%)	26 (4.62%)	1 (0.18%)

**Table 3:** Behaviors that increase exposure to harmful chemicals. EDC: endocrine-disrupting chemical

Question	Frequency n(%)				
How often do you…	Always	Often	Sometimes	Rarely	Never
heat food in a microwave using plastic containers?	67 (11.90%)	71 (12.61%)	78 (13.85%)	116 (20.60%)	231 (41.03%)
eat food cooked in non-stick pots and pans?	203 (36.06%)	192 (34.10%)	112 (19.89%)	42 (7.46%)	14 (2.49%)
consume canned food?	26 (4.62%)	124 (22.02%)	196 (34.81%)	172 (30.55%)	45 (7.99%)
use air fresheners and fragrances?	130 (23.09%)	197 (34.99%)	129 (22.91%)	97 (17.23%)	10 (1.78%)
use cosmetics, lotions, shampoo, or nail polish that are not labeled as EDC-free?	254 (45.12%)	151 (26.82%)	99 (17.58%)	50 (8.88%)	9 (1.60%)
consume food wrapped in plastic or plastic water bottles?	282 (50.09%)	176 (31.26%)	59 (10.48%)	39 (6.93%)	7 (1.24%)
use pesticides in the garden and at home?	41 (7.28%)	90 (15.99%)	168 (29.84%)	191 (33.93%)	73 (12.97%)
eat take-out food from coated cardboard or plastic containers?	298 (52.93%)	148 (26.29%)	81 (14.39%)	19 (3.37%)	17 (3.02%)
take the thermal receipt after purchases?	262 (46.54%)	134 (23.80%)	84 (14.92%)	23 (4.09%)	60 (10.66%)

Overall potential risk of exposure and awareness

The average score for potential EDC exposure was 32.78 (out of a maximum of 60). Scores ranged from 13 to 54 points. The largest group (85.26%, n=480) fell into the moderate potential exposure category. Our results identified a very low proportion (4.26%, n=24) at potential high risk of EDC exposure according to their reported daily habits. Among those at potential high risk, the majority (95.83%, n=23) were male participants. Approximately 25% (n=138) of participants reported prior knowledge of EDCs. Among those aware, only 20.29% (n=28) actively practiced behaviors to minimize EDC exposure.

**Figure 1:**
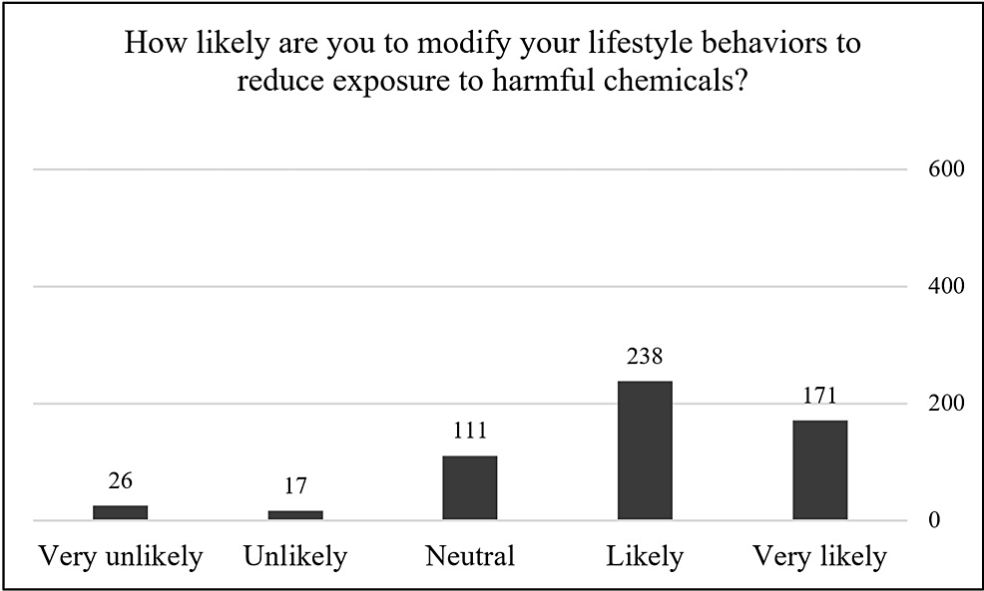
Likelihood of the participants following exposure reduction approaches (n=563). The data has been presented as frequencies (n).

More than 72% (n=409) of the participants stated that they are likely or very likely to follow lifestyle measures to reduce exposure to harmful substances, and only 4.6% (n=26) said they are very unlikely to make any changes in their daily habits. Figure 1 displays the participants' responses to the likelihood question.

Table 4 demonstrates the association between the participants' characteristics and their potential risk of exposure to EDCs. Statistically significant associations (p-values <0.05) were identified between the potential risk of EDC exposure and age, gender, area of residence, and education level, with p-values <0.05 (0.001*, 0.001*, 0.001*), respectively. No significant association (p=0.750) was found between the potential risk of EDC exposure and education level.

**Table 4:** The association between socio-demographic information and the potential level of exposure to EDCs. The data has been presented as frequencies (n) and percentages (%). *Significant at p<0.05 level. EDC: endocrine-disrupting chemical

		Potential risk of exposure			
Variables	Category	Low (0-21)	Moderate (21-40)	High (41-60)	P-value
Age in years	Less than 18	2 (5.41%)	31 (83.80%)	4 (10.81%)	<0.001*
	18-25	20 (7.30%)	246 (89.78%)	8 (2.92%)	
	26-35	17 (12.88%)	107 (81.06%)	8 (6.06%)	
	36-45	3 (5.77%)	48 (92.31%)	1 (1.92%)	
	46-55	8 (17.78%)	34 (75.56%)	3 (6.67%)	
	56-65	9 (40.90%)	13 (59.10%)	0 (0.00%)	
	More than 65	0 (0.00%)	1 (100.00%)	0 (0.00%)	
Gender	Female	31 (10.51%)	263 (89.15%)	1 (0.34%)	<0.001*
	Male	28 (10.45%)	217 (80.97%)	23 (8.58%)	
Area of residence	Central region	25 (11.85%)	180 (85.31%)	6 (2.84%)	<0.001*
	Southern region	8 (6.61%)	106 (87.60%)	7 (5.79%)	
	Western region	3 (3.30%)	82 (90.10%)	6 (6.60%)	
	Northern region	19 (21.33%)	52 (69.33%)	4 (5.33%)	
	Eastern region	4 (6.15%)	60 (92.30%)	1 (1.54%)	
Education level	Doctorate	1 (50.00%)	1 (50.00%)	0 (0.00%)	0.750
	Masters	2 (9.09%)	19 (86.36%)	1 (4.55%)	
	Bachelor’s	21 (8.50%)	215 (87.04%)	11 (4.45%)	
	Diploma	10 (4.05%)	49 (79.03%)	3 (4.84%)	
	High school	21 (10.45%)	171 (85.05%)	9 (4.50%)	
	Middle school	4 (14.29%)	24 (85.71%)	0 (0.00%)	
	Elementary school	0 (0.00%)	1 (100.00%)	0 (0.00%)	
Familiarity with EDCs	Yes	28 (20.29%)	107 (77.54%)	3 (2.17%)	<0.001*
	No	31 (7.29%)	373 (87.77%)	21 (4.94%)	

## Discussion

This study aims to identify lifestyle and behavioral habits and their relationship to EDC exposure among the people of Saudi Arabia. This study was conducted among a total of 563 participants from the population of Saudi Arabia, with a gender distribution nearly equal. The majority of the participants were in the age group of 18-25 years and held a bachelor's degree. Of these participants, the largest portion of them are from the central region. 

The study highlights various behaviors participants adopt to minimize exposure to harmful chemicals. Around 49.73% always wash new items before use, 47.07% regularly ventilate their homes, 65.01% consistently wash and peel produce, and 49.02% use damp cloths to clean dust. Additionally, 14.03% always consume organic food, and 46.00% regularly wash their hands before eating. These actions range from frequent practices, such as washing new products and ventilating homes, to less common behaviors like consuming organic food, illustrating varying levels of commitment to reducing exposure to harmful substances.

Furthermore, Table 3 reveals varying frequencies of behaviors that might influence chemical exposure. Notably, 41.03% rarely or never heat food in plastic containers using a microwave, which was a positive behavior. This contrasts with a previous study conducted in 2016 [15] regarding the use of disposable plastic containers by medical science students in Northeastern Iran, which discovered that although the majority of these students were aware of the negative environmental effects of these containers, there was a significant gap between their awareness and their actual behavior. Many students persisted in using disposable plastics even after becoming aware of the problems.

However, in our study, 36.06% of the participants always eat food cooked on non-stick cookware, which could increase chemical exposure. Canned food consumption varies, with 34.81% eating it sometimes, 26.02% always, and 30.55% rarely, indicating potential exposure to bisphenol A. Air fresheners and fragrances are always used by 23.09% and often by 34.99%, posing a risk due to volatile organic compounds (VOCs). Over 45% always use personal care products not labeled as EDC-free, increasing potential exposure to EDCs. Pesticide use is moderate, with 29.84% using them sometimes, and thermal receipts, containing bisphenol A, are always taken by 46.54%. About half (50.09%) of the participants always consume food wrapped in plastic or plastic bottles, and 52.93% frequently eat take-out from coated containers, both common sources of chemical exposure. With an emphasis on issues related to chemicals and health, the Du Preez et al. [16] study examined young individuals' knowledge and behaviors about plastic food packaging in South Africa. It was shown that although a large number of participants were aware of the possible health risks that may arise from plastic packaging, their conduct was inconsistent. Due to its price and ease of use, a sizable percentage of young adults continued to use plastic food packaging despite reservations.

In this study, we found statistically significant associations between age and the potential risk of exposure to EDCs. Out of the 563 people participating in this study, we found 246 of those aged 18-25 had a moderate potential risk of exposure to EDC, as they represent the largest portion. We can explain that for several possible reasons, including that this age group may have higher rates of personal care product use, which can be contained in EDC [17]. Also, changes in diet (such as consumption of canned and inorganic food) and living environment (such as living in industrial cities) may contribute to increasing the possibility of exposure to these EDCs.

Of all the participants in this study, females represent the largest portion of participants at moderate potential risk of exposure to the EDC. We found that the gender of participants in this study was statistically significant and there are associations between being female and an increased EDC potential risk of exposure. This may be due to several factors, including personal care products and dietary patterns. Similarly, the lifestyle choices and levels of exposure to phthalates and bisphenol A among women in Northern Italy who are of reproductive age were investigated in the Di Napoli et al. research [18]. The study discovered that elevated levels of exposure to these chemicals were associated with specific lifestyle habits, including food and the use of personal care products.

Also, there are statistically significant associations between the area of residence and the potential risk of exposure to EDCs. The participants in this study from the central region represent the largest portion of the participants who had a moderate potential risk of exposure to EDC. There are factors that may contribute to explaining this result, including the fact that the majority of the participants in this study are from the central region, which represents the highest response rate (37.48%, n=211). This may play an important role in explaining this result. Also, the central region represents modern cities, and because of this, it may contribute to the increased exposure of people from this region to EDC more than the rest of the regions in Saudi Arabia.

It's interesting to note that there was no evidence in our study that education level and the chance of being exposed to EDCs were significantly correlated. The previous study, conducted by El-Sayed et al. [19], examined the impact of an educational intervention on mothers' attitudes, knowledge, and behaviors toward the proper use of plastic containers in minimizing their exposure to EDCs. In contrast to our study, the mothers' levels of education were shown to be strongly correlated to exposure-related behaviors and awareness of EDC.

Limitations and recommendations

While this study offers valuable insights, it is important to acknowledge some limitations. Firstly, the study relied on the HEAL [13] and EWG [14] guidelines for EDC exposure reduction to formulate the questionnaire. These guidelines cover a list of common daily in-home behaviors. However, exposure to EDCs can occur through many other routes that were not covered in the questionnaire, some of which are inevitable. Moreover, the questionnaire asks the participants to report the frequency of those behaviors within the last few months, which may predispose them to recall bias in addition to not being able to report an exact dose of exposure. Therefore, the study can only provide an approximate and qualitative description of the current behaviors and the reflected potential risk. Additionally, while our cross-sectional study determined some associations between the participants' characteristics and the potential exposure levels to EDCs, it was not able to assess causal relationships. Lastly, the presented study only included Saudi participants, and further studies may assess the association between nationality and behavior. Recommendations for future research include expanding the behaviors list to include more specific behaviors in and outside the home, assessing the link between awareness and behaviors directly, and measuring the quantitative EDC levels of the participants to provide more precise results.

## Conclusions

This study demonstrates a range of behavioral habits associated with exposure to endocrine disruptors in the Saudi Arabian general population. Most of the subjects were classified as having a moderate potential risk of exposure. A small percentage, mostly consisting of men, were at a possibly high risk of EDC exposure based on their reported daily activities. Age, gender, and area of residence were shown to be significantly correlated to behavioral habits in our study. The majority of our study participants showed a positive attitude when asked about their willingness to modify their habits.

Our results highlight the need for a set of educational programs and public health campaigns to supply the public with the information needed to establish exposure reduction strategies. Reducing chemical exposure lowers the potential hazards associated with higher EDC doses and ensures human well-being by improving health outcomes.
